# Virulence *Vs.* Immunomodulation: Roles of the Paracoccin Chitinase and Carbohydrate-Binding Sites in *Paracoccidioides brasiliensis* Infection

**DOI:** 10.3389/fmolb.2021.700797

**Published:** 2021-08-31

**Authors:** Nayla de Souza Pitangui, Fabrício Freitas Fernandes, Relber Aguiar Gonçales, Maria Cristina Roque-Barreira

**Affiliations:** Department of Cell and Molecular Biology and Pathogenic Bioagents, Ribeirão Preto Medical School, University of São Paulo, Ribeirão Preto, São Paulo, Brazil

**Keywords:** fungal chitinases, paracoccin, virulence factor, immunomodulatory agent, chitinase site, lectin site, P. brasiliensis

## Abstract

Paracoccin (PCN) is a bifunctional protein primarily present in the cell wall of *Paracoccidioides brasiliensis*, a human pathogenic dimorphic fungus. PCN has one chitinase region and four potential lectin sites and acts as both a fungal virulence factor and an immunomodulator of the host response. The PCN activity on fungal virulence, mediated by the chitinase site, was discovered by infecting mice with yeast overexpressing PCN (PCN-ov). PCN-ov are characterized by increased chitin hydrolysis, a narrow cell wall, and augmented resistance to phagocytes' fungicidal activity. Compared to wild-type (wt) yeast, infection with PCN-ov yeast causes a more severe disease, which is attributed to the increased PCN chitinase activity. In turn, immunomodulation of the host response was demonstrated by injecting, subcutaneously, recombinant PCN in mice infected with wt-*P. brasiliensis*. Through its carbohydrate binding site, the injected recombinant PCN interacts with Toll-like receptor 2 (TLR2) and Toll-like receptor 4 (TLR4) N-glycans on macrophages, triggers M1 polarization, and stimulates protective Th1 immunity against the fungus. The PCN-treatment of wt yeast-infected mice results in mild paracoccidioidomycosis. Therefore, PCN paradoxically influences the course of murine paracoccidioidomycosis. The disease is severe when caused by yeast that overexpress endogenous PCN, which exerts a robust local chitinase activity, followed by architectural changes of the cell wall and release of low size chito-oligomers. However, the disease is mild when exogenous PCN is injected, which recognizes N-glycans on systemic macrophages resulting in immunomodulation.

## Introduction

Fungal chitinases (EC 3.2.1.14) hydrolyse chitin, a β-(1,4)-N-acetyl D-glucosamine homopolymer that is a primary constituent of the fungi cell wall (Puccia et al., 2011). Chitin degradation enables the remodeling and constant plasticity required for cell wall architecture of yeast and filamentous fungi (Adrangi and Faramarzi, 2013; Langner and Gohre, 2016). The identification of fungal chitinases is based on their similarities with the 18 families of bacterial or plant chitinases, whose classifications are well established in the literature (Takaya et al., 1998). Fungal chitinases have the highest homology with class III plant chitinases (Hayes et al., 1994), which frequently include carbohydrate-binding modules (CBM). In fungi, the reported CBMs are 1) CBM18 has a chitin-binding domain, 2) CBM1 has a cellulose- and chitin-binding domain; and 3) CBM50 has a peptidoglycan- and chitin-binding domain, also known as LysM (Seidl, 2008). Chitinases are classified as glycosyl hydrolases (GH) based on the amino acid sequence similarity of the catalytic domains, the chitinases are distributed in families 18, 19, and 20 of GH (Duo-Chan, 2006). Fungal chitinases belong mainly to family 18 and play relevant roles in nutrition, parasitism, morphogenesis, immunity, and autolysis (Karthik et al., 2014).

Genes encoding chitinases have been identified and characterized in yeast, such as *Saccharomyces cerevisiae*, *Candida albicans* (Dunkler et al., 2005), and *Kluyveromyces lactis*. In filamentous fungi, there are chitinases in the genera *Trichoderma*, *Penicillium*, *Lecanicillium*, *Neurospora*, *Mucor*, *Beauveria*, *Lycoperdon*, *Aspergillus*, *Myrothecium*, *Conidiobolus*, *Metharhizium*, *Stachybotrys*, *Agaricus*, and *Ashbya* (Duo-Chuan, 2006; Karthik et al., 2014; Dunkler et al., 2008). In dimorphic fungi, chitinase genes have been reported in *Coccidioides immitis* (Cole and Hung, 2001), *Histoplasma capsulatum* (Goughenour et al., 2021), and *Paracoccidioides brasiliensis* (dos Reis Almeida et al., 2010; Santana et al., 2012; Fernandes et al., 2017).

In the last decade, we identified and characterized a *P. brasiliensis* protein, paracoccin (PCN), which has both lectin (Coltri et al., 2006; Ganiko et al., 2007) and chitinase (dos Reis Almeida et al., 2010) properties. When examined from a fungus-host relationship perspective, PCN was revealed to play roles that are somehow paradoxical. Soluble recombinant PCN, administered to *P. brasiliensis* infected hosts, acts as an immunomodulatory agent that favors protection against the fungus (Alegre-Maller et al., 2014; Alegre et al., 2014). In contrast, endogenous PCN, expressed by the infecting fungus itself, acts as a *P. brasiliensis* virulence factor (Fernandes et al., 2017; Gonçales et al., 2021) that aggravates the infection in a murine model. The first *P. brasiliensis* protein explored concerning this functional duality in the host-pathogen relationship was a surface glycoprotein of 43 kDa, known as gp43, which acts as a *P. brasiliensis* adhesin that binds laminin and fibronectin (Vicentini et al., 1994). In addition, a 15-amino acid peptide of gp43 (QTLIAIHTLAIRYAN), designated as P10, was shown to protect a *P. brasiliensis* infected host against the development of paracoccidioidomycosis (PCM). Additionally, P10 was effective in treating a well-established disease (Magalhães et al., 2012).

This article describes the structural organization of the PCN catalytic and putative carbohydrate-binding regions and the paradoxical effects of each type of region, acting as a *P. brasiliensis* virulence factor and an immunomodulatory agent that induces protection against PCM.

## Paracoccin Structural Features

**FIGURE 1:**
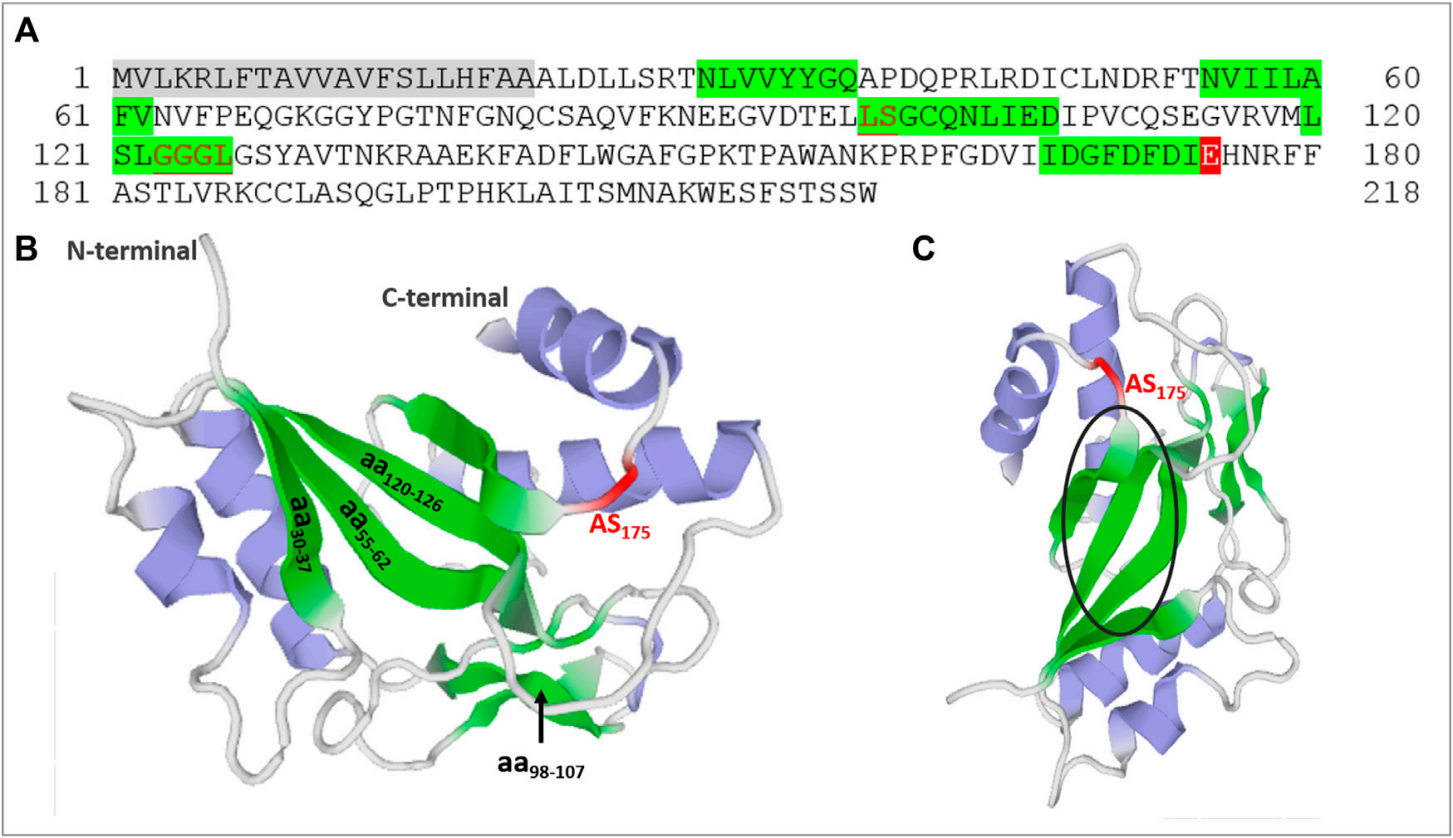
Identification of catalytic and potential carbohydrate-binding regions in paracoccin (PCN). **(A)** The PCN amino acid residue sequence (PADG_03,347) from *P. brasiliensis* used in protein domain analyzes. The gray is the signal sequence, and the red is the catalytic site of the family 18 chitinases domain. Glutamic acid at position 175 is associated with the catalytic site of the chitinase domain. The green indicates regions of the PCN likely to bind to carbohydrates. The underlined red letters were chito-oligosaccharide-binding regions determined by Prosite software. **(B)** Molecular model of PCN with potential carbohydrate-binding regions and chitinase active site (AS) identified in green and red. **(C)** Molecular model of PCN from another angle (90^o^) with a black circle highlighting the beta sheet tertiary structure in the three-dimensional molecule.

In a search for protein regions responsible for the PCN activities, we performed amino acid sequence analysis on the annotated PADG_03,347 sequence (Alegre et al., 2014) retrieved from fungi database site (https://fungidb.org/fungidb/) and examined it with the aid of ExPASy software’s (http://www.cbs.dtu.dk/services/SignalP/; https://prosite.expasy.org/). We verified that PADG_03,347 encompassed a signal peptide whose cleavage site was in amino acid residues 22 and 23 and a domain homologous to glycoside hydrolase 18 (GH18) family of chitinases (29–218) whose glutamic acid at position 175 was associated with the catalytic site of the chitinase site (Figure 1A). Since PCN activity depends on the recognition of N-glycans linked to Toll-like receptor 2 (TLR2) and Toll-like receptor 4 (TLR4) (Alegre-Maller et al., 2014), we investigated whether the deduced protein primary structure contained potential carbohydrate-binding sites. Amino acid residue sequences of lectins from different organisms, including fungal proteins, already characterized as having carbohydrate-binding sites, were collected from databases and used for comparison with the amino acid sequence of PCN. From analysis at Clustal Omega (https://www.ebi.ac.uk/Tools/msa/clustalo/), we identified the regions between amino acids 30–37, 55–62, 98–107 and 120–126 containing aligned nonpolar amino acid residues, which are frequently involved in carbohydrate recognition. Drickamer and Dood (1999) reported this characteristic set of hydrophobic residues in carbohydrate recognition domains (CRDs) of lectins. More recently, polar amino acids were identified as necessary for the protein-carbohydrate interaction, which involves hydrogen bonding with sugar hydroxyl groups, CH–π interactions with aromatic amino acid side chains, and coordination to calcium ions (Hudson et al., 2015; McMahon et al., 2020). The PCN analysis has also revealed the presence of amino acid residues with polar properties that could potentially contribute to the interaction with carbohydrates. A second PCN analysis, using the Prosite software (https://prosite.expasy.org/), determined two chitooligosaccharides binding regions in amino acid residues 98–99 and 123–126. These observations suggest four potential carbohydrate-binding regions on PCN and one catalytic site.

We also obtained the three-dimensional molecular model of PCN by submitting its amino acid sequence to the Swiss-model software (https://swissmodel.expasy.org/). The structure used as a template was ChiA1 of *Aspergillus fumigatus* in complex with acetazolamide (PDB 2XTK) with 47.47% identity. Figure 1B,C show the localization of the carbohydrate-binding regions predicted from the sequence analysis and the active chitinase site. It is worth highlighting the position of the CRDs converged at the same region of the molecule’s three-dimensional structure.

## Recombinant Paracoccin Induces Protective Immunity Against Fungal Infection

Because of their carbohydrate-binding sites, PCN interacts with GlcNAc monomer and homopolymers (Ganiko et al., 2007; dos Reis Almeida et al., 2010), as well as with GlcNAc-containing N-glycans, such as the ones found in the glycoproteins laminin (Ganiko et al., 2007), TLR-2, and TLR-4 (Alegre-Maller et al., 2014; Freitas et al., 2016). The *in vitro* demonstration that PCN interaction with TLRs on macrophages induces cell activation and signalling for pro-inflammatory cytokine production, such as IL-12, has encouraged investigations on immunomodulatory property of PCN (Alegre-Maller et al., 2014). Simultaneously, some lectins were isolated from human pathogenic fungi (reviewed by Gallegos et al., 2014). Several reports that lectins from pathogens provide adherence for the pathogen to host epithelial cells suggested that the lectins could account for relevant biological activities and make them attractive targets for developing novel drugs (Houser et al., 2013). Despite this, only a few lectins from pathogenic fungi have been studied in detail until now. In an experimental model of human paracoccidioidomycosis, when recombinant PCN is injected subcutaneously in mice, the course of the disease is milder. Similarly, murine toxoplasmosis is also modified by injecting the recombinant lectins from *Toxoplasma gondii,* microneme proteins 1 (MIC-1) and 4 (MIC-4) (Lourenço et al., 2006).

PCN administration to *P. brasiliensis* infected mice confers protection against paracoccidioidomycosis through a mechanism primarily triggered by PCN interaction with N-glycans of TLR2 and TLR4 on the surface of macrophages. Detailed studies on TLR2 have identified which N-glycans mediate responses to PCN stimulus. Its effects on transfected cells expressing only a certain TLR2 glycomutant, or a combination of glycomutants, were compared to that exerted-on cells expressing the wt-receptor (Alegre-Maller et al., 2014). We verified that the presence of the fourth N-glycan, among the four TLR2 N-glycans, was enough to induce cell activation. Interestingly, site 4 of TLR2 N-glycosylation is the most conserved and least accessible among all TLR2 N-glycosylation sites (Ricci-Azevedo et al., 2017). All the TLR2 glycomutants unresponsive to PCN were functional, i.e., mediated cell activation, when stimulated by a classical TLR2 agonist, Pam3CSK4. These findings demonstrated that PCN activity depends on receptor glycosylation. The responsive macrophages responsive to PCN become M1 polarized cells (Freitas et al., 2016) that release cytokines, such as IL-12, which stimulate the development of T helper 1 (Th1) cells (Coltri et al., 2006; Alegre-Maller et al., 2014; Alegre et al., 2014; Freitas et al., 2016). The Th1 cells release IFN-γ and are protective against most systemic fungal diseases (Verma et al., 2014). The co-production of high and persistent levels of TNF-α (Coltri et al., 2006) and nitric oxide also contributes to host protection by increasing vascular permeability and tissue accumulation of effector macrophages, with augmented fungicidal activity. Our studies have demonstrated that, by interacting with TLRs N-glycans on macrophages, PCN acts as an immunomodulatory agent to drive the host immune response to a Th1 protective axis. As an efficient immunomodulator, PCN became the focus of further studies designed to elucidate its role in fungal biology and pathogenesis.

## Endogenous Paracoccin Favors Fungus Virulence and Pathogenicity

PCN distribution in the cell wall of *P. brasiliensis* hyphae, transition forms, and mature yeast was detected by confocal microscopy using anti-paracoccin antibodies (Ganiko et al., 2007; Oliveira et al., 2017). Fluorescent labelling of PCN occurred predominantly in structures related to fungal growth, such as hyphae tips, chlamydospores of differentiating hyphae, and the budding regions of yeast, supporting the hypothesis that PCN has a function in fungal growth and dimorphic transformation (Ganiko et al., 2007; Oliveira et al., 2017).

To explore the role of PCN in fungal biology and pathogenesis, we have genetically modified the PCN expression in *P. brasiliensis* yeast. The difficulties known to be inherent to *P. brasiliensis* molecular manipulation were overcome with the indispensable collaboration of Dr. Fernando Rodrigues (School of Health Sciences, University of Minho, Braga, Portugal) using the technique based on *Agrobacterium tumefaciens* mediated transformation (ATMT). We successfully modified the gene PADG_03,347 (Gene ID: 22582669) and generated PCN knocked-down (kd-PCN) (Fernandes et al., 2017) and PCN overexpressing (ov-PCN) (Gonçales et al., 2021) yeast. Levels of PCN mRNA expression were reduced 60% in kd-PCN, and augmented 7-fold in ov-PCN compared to wt-yeast. Consistently, modified PCN protein detection was determined by confocal microscopy and chitinase activity in kd-PCN and ov-PCN yeast.

kd-PCN yeast are very agglomerated, a characteristic attributed to impaired daughter cell detachment due to PCN deficiency. Other authors verified yeast agglomeration in other fungal species where the genes coding for chitinase had been deleted (Kuranda and Robbins, 1991; Dunkler et al., 2005). The decreased PCN expression was also related to *P. brasiliensis* yeast’s incapacity to transition to mycelium. This process primarily depends on coordinated cell wall remodelling chitinase activity (Bastos et al., 2007). In contrast, overexpression of the gene PADG_03,347 allowed a robust separation of the budding yeast from the mother cells. These isolated transformed yeasts displayed reduced cell size and accelerated yeast-to-hypha transition. Increased expression of PCN is readily detectable in the cell wall of ov-PCN yeast, whose chitin content is diminished. Compared to the wt-yeast, the ov-PCN yeast cell wall was about 6 times thinner and was accompanied by a thick outer layer of a fibrillar material, compatible with the presence of a substantial amount of GlcNAc oligomers. Because the ov-PCN total carbohydrate content was higher than that detected in wt-yeasts and PCN has chitinase activity, we examined the particles of β-1,4-linked GlcNAc oligomers captured by affinity to WGA from the culture supernatants of ov-PCN and wt-yeasts. The particles isolated from wt-PCN varied widely in size and ranged up to 240 nm^2^, while those from ov-PCN were smaller and had a narrower size distribution ranging up to 60 nm^2^. PCN overexpression, associated with increased chitinase-activity, leads to greater hydrolysis of the yeast's cell wall chitin into smaller chito-oligomers, a reduced β-1,4-GlcNAc content, and diminished cell wall thickness (Gonçales et al., 2021). These alterations culminate in altering the cell wall architecture.

Functional *in vitro* assays have shown that PCN-silencing does not affect the adherence or phagocytosis of *P. brasiliensis* yeasts by host cells but renders the yeast more susceptible to the macrophages fungicidal activity (Fernandes et al., 2017). PCN-overexpressing, in turn, augments yeast phagocytosis by macrophages and renders the yeast more resistant to macrophages fungicidal mechanisms (Gonçales et al., 2021).

**FIGURE 2:**
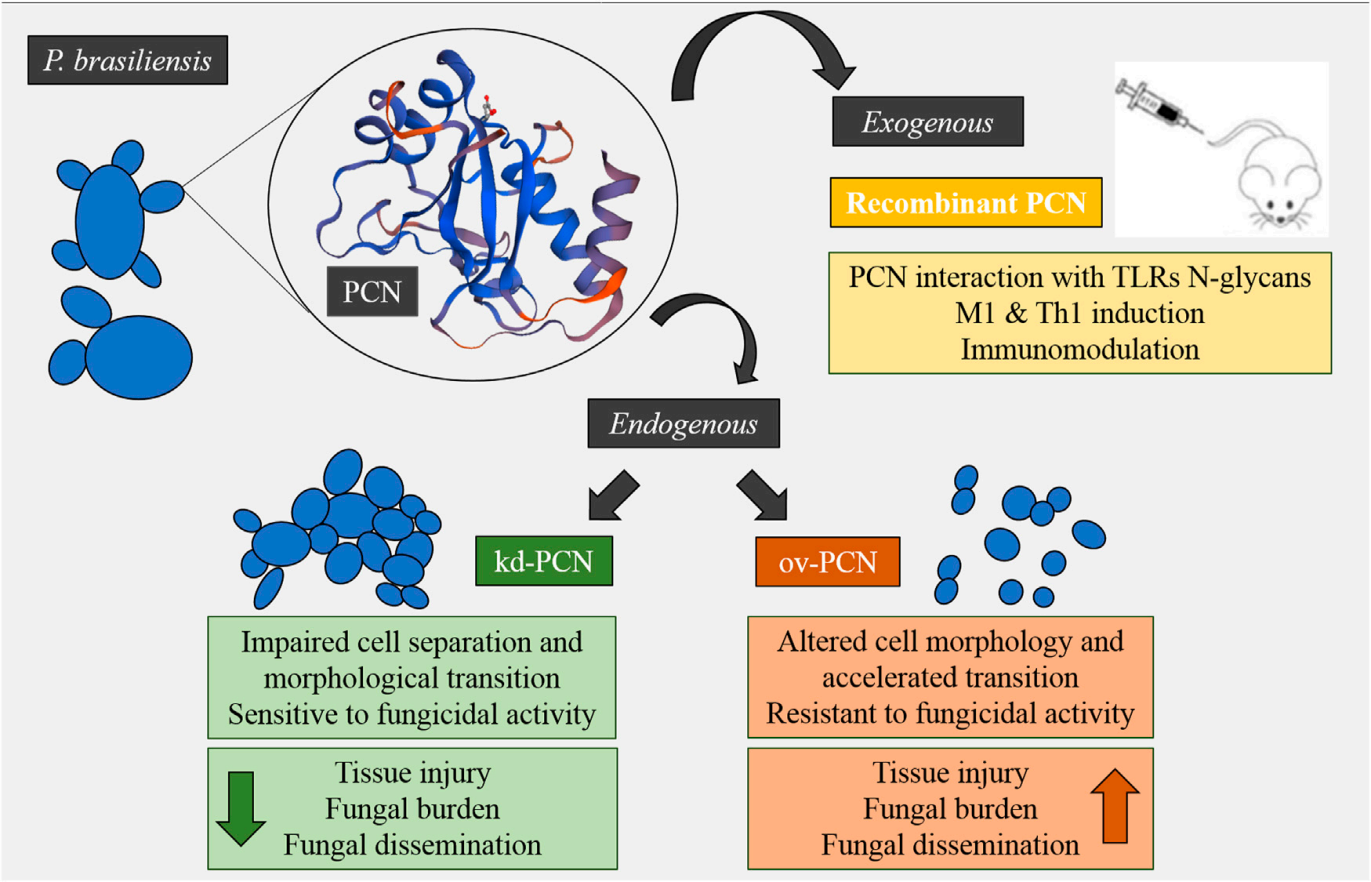
Concise model of the effect of exogenous and endogenous PCN on fungal virulence and modulation of the host’s immune response.

Mice infected with kd-PCN yeast, compared to wt-yeast, had a mild pulmonary disease, manifested by a low pulmonary fungal burden, few focal inflammatory lesions, and reduced mortality rates (Fernandes et al., 2017). In contrast, mice infected with ov-PCN yeast, compared to wt-yeast, developed a severe systemic disease accompanied by elevated fungal load, extensive and coalescent lung granulomas, and high mortality rates (Gonçales et al., 2021) (Figure 2).

The contrasting disease patterns produced by kd-PCN and ov-PCN yeast are consistent with the verified *in vitro* features of the transformed yeast, in the sense that kd-PCN yeast are susceptible (Fernandes et al., 2017) and ov-PCN yeasts are resistant to the fungicidal activity of phagocytes (Gonçales et al., 2021).

The host protection conferred by injection of exogenous PCN in *P. brasiliensis* infected mice was the focus of our initial studies on PCN (Coltri et al., 2006; Alegre et al., 2014; Alegre-Maller et al., 2014; Freitas et al., 2016). These observations lead us to consider PCN as just an immunomodulatory agent that favors host resistance to *P. brasiliensis* infection (Ricci-Azevedo et al., 2017). Therefore, the results obtained with mice infected with kd-PCN and ov-PCN yeast (Fernandes et al., 2017; Gonçales et al., 2021), seemed to conflict with our assumptions. We expected that ov-PCN yeast would cause an auto-resolving illness due to their high content of an agent able to drive host immunity toward a Th1 response. We attributed the mechanism of the protection induced by PCN to the interaction of its CRDs with TLRs N-glycans which leads to phagocyte activation with the subsequent production of TNF-α, NO, and IL-12. The culmination of the phagocyte activation is the killing of yeast by macrophages and T cells. Nonetheless, yeast infection with increased endogenous PCN-expression brought no beneficial effect to the host. On the contrary, as already mentioned, mice infected with ov-PCN yeast became severely ill with a high fungal burden, coalescent pulmonary granulomatous lesions, and a high mortality rate (Gonçales et al., 2021). This disease configuration was further explained by the action of the chitinase domain of endogenous PCN on the cell wall chitin, increasing the yeast resistance to killing and overcoming the immunomodulatory effect triggered by the eventual binding of PCN lectin sites to host cells' TLRs. The abundant insoluble PCN, localized in the yeast cell wall, does not have easy access to macrophages, whose TLRs N-glycans would be targeted by the PCN lectin domain, as verified for soluble PCN. In the fungal cell wall, PCN intermingles with chitin, a substrate efficiently cleaved by the PCN enzymatic region, allowing the formation of a broad external layer of chito-oligomers. We postulate that the small yeast-derived chito-oligomers, once released, can exert a regulatory role on cytokines production by neighbouring macrophages.

Chitin particles are known to induce a wide range of polarized macrophage activation, depending, at least in part, on particle size. Particles larger than macrophages are difficult to phagocytose and are frequently inert in terms of inducing macrophage activation. Differently, microparticles, possessing at least 6 GlcNAc oligomers, are capable of being phagocytosed and activate TLR2 on macrophages and induce their M1 polarization, an event identified by prominent TNF-α production. Smaller chitin particles interact with other pattern recognition receptors and induce M2 macrophages, which produce anti-inflammatory cytokines, such as IL-10. When this occurs in the early phases of the fungal infection, it dampens the Th1 protective immunity response (Costa et al., 2013). Therefore, chito-oligomers can stimulate antagonistic responses depending on their size (Da Silva et al., 2009; Wagener et al., 2014; Fuchs et al., 2018). The severe disease developed by infected mice with ov-PCN yeasts may be due to the already cited resistance to fungicidal mechanisms and IL-10 production, dampening the host's Th1 protective immune response. We expect to answer this question through experiments that are in progress in our laboratory.

Although there are still some mechanistic questions to be answered, previous studies have provided strong evidence that the PCN lectin and enzymatic sites exert opposite effects on the course of *P. brasiliensis* infection.

## Outlook

The observations reviewed herein show that administering the soluble recombinant *P. brasiliensis* component PCN to infected mice benefits the host through actions mediated by the PCN lectin sites. In contrast, augmented expression of endogenous PCN in the infective transformed yeast favors the fungal virulence and pathogenicity through actions mediated by the PCN chitinase site.
